# Surveillance for Shiga Toxin–producing *Escherichia coli,* Michigan, 2001–2005

**DOI:** 10.3201/eid1302.060813

**Published:** 2007-02

**Authors:** Shannon D. Manning, Robbie T. Madera, William Schneider, Stephen E. Dietrich, Walid Khalife, William Brown, Thomas S. Whittam, Patricia Somsel, James T. Rudrik

**Affiliations:** * Michigan State University, East Lansing, Michigan, USA; † Michigan Department of Community Health, Lansing, Michigan, USA; ‡ Sparrow Health System, Lansing, Michigan, USA; § Detroit Medical Center, Detroit, Michigan, USA

**Keywords:** *Escherichia coli*, surveillance, Shiga toxin, epidemiology, dispatch

## Abstract

A surveillance system used different detection methods to estimate prevalence of Shiga toxin–producing *Escherichia coli* during 2003–2005 and 2001–2002. More non-O157 serotypes were detected by enzyme immunoassay than by evaluation of non-sorbitol–fermenting *E. coli* isolates. We therefore recommend use of enzyme immunoassay and culture-based methods.

Infection with Shiga toxin–producing *Escherichia coli* (STEC) is a frequent cause of gastrointestinal disease, particularly among children and elderly persons (*1*). Detection of O157 STEC by culture relies primarily on sorbitol MacConkey agar (SMAC) (*2*) because O157:H7 strains cannot rapidly ferment sorbitol (*3*). In contrast, using culture to detect sorbitol-fermenting O157 (*4*) and non-O157 serotypes is problematic because on SMAC these strains are indistinguishable from other *E. coli*. Consequently, whether the predominance of STEC O157 in disease reflects actual differences in pathogen prevalence or a bias associated with detection is unclear. We therefore sought to determine whether STEC prevalence, particularly of non-O157 serotypes, increased when enhanced detection methods were used.

## The Study

The Michigan Department of Community Health implemented a sentinel surveillance system to evaluate blood-containing stool samples from 20 laboratories during April 2003–October 2005 and all stool samples from 2 hospitals during July 2004–October 2005. All suspect non-sorbitol–fermenting *E. coli* from the remaining laboratories were also examined.

The samples, transported in C&S transport medium (Medical Chemical Corporation, Torrance, CA, USA), were screened for Shiga toxin (Stx) by enzyme immunoassay (EIA) (Meridian BioScience, Cincinnati, OH, USA) after enrichment with gram-negative broth (Remel, Lenexa, KS, USA). EIA is sensitive and specific but cannot detect the Stx2e variant (*5*), and *Pseudomonas aeruginosa* can produce false-positive results (*6*). Samples were cultured on SMAC (Remel) and cefixime-tellurite SMAC (*7*), and samples from the 2 hospitals were tested for occult blood (Beckman Coulter, Fullerton, CA, USA) before EIA testing. Serotyping (Statens Serum Institute, Copenhagen, Denmark; BD Difco, Franklin Lakes, NJ) and real-time PCR for *stx1,2* genes (*8*) were performed on strains that had positive EIA results, suspect non-sorbitol–fermenting *E. coli*, and multiple colonies of sorbitol-fermenting (SF) strains that had positive EIA results. For some samples, the EIA result was negative but NSF *stx*-positive colonies were detected on SMAC, which indicated a false-negative EIA result. Epidemiologic data were obtained for STEC-positive patients.

During the 5 years studied, 438 STEC were isolated; 401 (92%) were O157. Prevalence over time did not differ (χ^2^ = 4.14, df = 4, p = 0.39). Similarly, overall prevalence of non-O157 serotypes during 2001–2002 and 2003–2005 did not differ (χ^2^ = 0.83, df = 1, p = 0.36). Most (70%) STEC isolates were recovered between June and October from heavily populated areas (Figure). No SF O157 were recovered.

**Figure:**
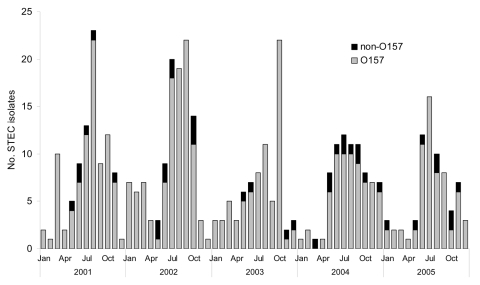
Frequency of isolation of Shiga toxin–producing *Escherichia coli* (STEC), Michigan, 2001–2005. Enhanced surveillance for STEC began in April 2003.

In 2001–2002, a total of 664 suspect NSF *E. coli* isolates were evaluated; 179 (27%) were O157 and 2 (0.3%) were non-O157 serotypes (Table 1). After enhanced surveillance began in 2003, a total of 852 suspect isolates were tested; 177 (21%) were O157 and 3 (0.4%) were non-O157 serotypes (Table 1). The remaining STEC (n = 28, 2001–2002; n = 49, 2003–2005) were detected by EIA. For 5 samples, EIA yielded a false-negative result but STEC were recovered from SMAC. During 2003–2005, 7 of the 49 STEC came from screening all 2,426 samples from the 2 hospitals; 359 (15%) of these samples contained occult blood. Among the 7 detected, 5 contained occult blood and 6 were O157. The remaining 42 (10%) STEC were found by screening 423 suspect samples from other laboratories; 18 (4%) were non-O157 serotypes. More non-O157 serotypes were detected (χ^2^ = 61.1, df = 1, p<0.00001) from 2003–2005 when EIA was used instead of the NSF *E. coli* isolate submission process. Among all 37 non-O157 serotypes isolated, O45:H2 (n = 10) and O26:H11 (n = 5) predominated.

**Table 1:** Evaluation of shiga toxin–producing *Escherichia coli* detection methods, Michigan, 2001–2005*

Detection method	Stool samples	Routine STEC surveillance†, no. (%)		Enhanced STEC surveillance, no. (%)		
		2001	2002	2003	2004	2005
Culture and PCR‡ for *stx* genes of suspect NSF *E. coli*	Total	327	337	249	317	286
	O157	85 (26)	94 (28)	63 (25)	58 (18)	56 (20)
	Non-O157	2 (0.6)	0 (0)	0 (0)	2 (0.6)	1 (0.3)
EIA of bloody or suspect samples	Total	N/A	N/A	209	141	73
	O157	4	11	11 (5)	7 (5)	6 (8)
	Non-O157	4	9	4 (2)	7 (5)	7 (10)
EIA of all samples	Total	N/A	N/A	N/A	1,405	1,021
	O157	N/A	N/A	N/A	5 (0.4)	1 (0.1)
	Non-O157	N/A	N/A	N/A	1 (0.07)	0 (0)

*STEC, Shiga toxin–producing *Escherichia coli*; NSF, non-sorbitol–fermenting; EIA, enzyme immunoassay. †In 2001 and 2002, EIA of bloody stools was performed by 2 large clinical laboratories in Michigan and was not part of the enhanced STEC surveillance system at the Michigan Department of Community Health. Therefore, the total number of EIAs performed is not known; the total number of EIA s submitted as positive for Stx is provided by serotype. ‡PCR for *stx1,2* genes was not initiated at the Michigan Department of Community Health until 2002. Before 2002, a DNA probe (Digene Signal Kit, Digene Diagnostic, Inc., Silver Spring, MD, USA) was used to detect STEC.

Epidemiologic data were available for up to 389 (89%) STEC patients, depending on the variable assessed. Most patients (62%) were Caucasian; ≈50% were female. Disease occurred mostly in persons ≤10 (27%), 11–18 (19%), and 19–30 (17%) years of age. Although disease frequency was lower (9%) in persons >65 years of age, these persons were more likely to be hospitalized than were persons ≤18 years of age, as were persons with bloody diarrhea or hemolytic-uremic syndrome (HUS) (Table 2). Among the 12 patients with HUS, 2 were infected with non-O157 serotypes O103:H2 and O76:H7, and 7 of the 12 HUS-associated strains were *stx2* only.

**Table 2:** Association of characteristics with infection by Shiga toxin–producing *Escherichia coli* among 389 of 438 patients for whom data were available, Michigan, 2001–2005*†

Characteristic	No. with characteristic	No. (%) hospitalized	OR (95% CI)
Demographics			
Age, y			
≤18	178	77 (43)	1.0
19–64	178	94 (53)	1.5 (0.95–2.28)
≥65	32	26 (81)	5.7 (2.09– 16.27)
Sex			
Female	204	105 (51)	1.0
Male	185	92 (50)	0.9 (0.61– 1.42)
Clinical symptoms			
Abdominal pain			
No	76	40 (53)	1.0
Yes	304	151 (50)	0.9 (0.54– 1.47)
Body aches			
No	323	161 (50)	1.0
Yes	57	30 (53)	1.1 (0.64– 1.96)
Chills			
No	313	155 (50)	1.0
Yes	66	36 (55)	1.2 (0.72– 2.08)
Diarrhea			
No	154	82 (53)	1.0
Yes	226	109 (48)	0.8 (0.54– 1.23)
Bloody diarrhea			
No	77	30 (39)	1.0
Yes	303	161 (53)	1.8 (1.10– 2.96)
HUS			
No	368	180 (49)	1.0
Yes	12	11 (92)	11.5 (1.47– 89.90)
Bacterial serotype and genes			
O157	360	181 (50)	1.0
Non-O157	29	16 (55)	1.2 (0.57– 2.60)
*stx1*	28	14 (50)	1.0
*stx2*	121	54 (45)	0.8 (0.33– 1.98)
*stx1,2*	239	128 (54)	1.2 (0.49- 2.70)

*OR, odds ratio; CI, confidence interval; HUS, hemolytic-uremic syndrome. †Some numbers do not add up to the total (n = 389) because of missing values (never >2.6%).

To adjust for factors associated with hospitalization, we fit a logistic regression model that included age and symptom variables in the model. The adjusted associations were similar to the crude associations. Hospitalization was more frequent for persons with bloody diarrhea (adjusted odds ratio [OR] 1.8, 95% confidence interval [CI] 1.04–3.08) and HUS (adjusted OR 16.0, 95% CI 2.00–127.47). Also, persons 19–64 (adjusted OR 1.6, 95% CI 1.05–2.59) and >65 (adjusted OR 6.6, 95% CI 2.57–17.15) years of age were hospitalized more frequently than persons ≤18 years of age.

## Conclusions

Enhanced detection methods did not significantly increase the year-to-year recovery of STEC. Overall, the observed STEC prevalence decreased slightly over time, similar to the national trend of an overall 42% decrease in STEC O157 incidence during 1996–2004 (*9*). This reduction is likely attributable to numerous factors, including heightened consumer awareness (*9*) and improved screening protocols during food production (*10*).

Enhanced surveillance did, however, enhance detection of non-O157 serotypes; 4.3% of EIA-positive stools were non-O157 compared with 0.5% of suspect NSF *E. coli*. Additionally, among the STEC found, 34 (48%) were non-O157 and 37 (52%) were O157 when EIA was used on suspect stools, compared with only 3 (1.6%) non-O157 and 177 (98.3%) O157 among NSF *E. coli*. Despite enhanced surveillance, STEC prevalence is probably still underestimated, particularly for non-O157 serotypes, because not all ill persons seek medical care and not all laboratories submit suspect stools for evaluation. Nevertheless, in 5 years, our surveillance identified 66 (15%) cases that would have been undetected by conventional methods; 31 (47%) were non-O157. Among those patients for whom data were available, 27 (42%) of 64 were <18 years of age, 22 (43%) of 51 were hospitalized, and 39 (76%) of 51 had bloody diarrhea. Although bloody stool and patient age are poor predictors of STEC infection (*11*), our analysis demonstrates that screening bloody stool samples improves detection of non-O157, and blood and older age are important predictors of more severe disease, which may be more costly if undetected.

Hospitalization of STEC patients with and without HUS costs an estimated US $30,307 and $4,061 per patient, respectively (*12*). Therefore, Michigan hospital costs associated with STEC infection likely exceeded $1,119,050 during 2001–2005, as 198 patients were hospitalized and 12 had HUS. Identification of each additional STEC case could have a substantial public health effect in that 1 case may lead to the recognition of an outbreak, which if detected early, could contribute to a cost savings as well as reduced STEC-associated illness. We estimated that the cost to detect each of the 66 additional cases using the EIA ($7 per test including labor) differed considerably when we evaluated screening of all stool samples ($2,426/per positive) versus suspect stool samples ($10/per positive).

No widely available test detects all STEC, and use of multiple methods is not cost-effective. Consequently, we recommend using EIA in conjunction with SMAC culture to recover isolates for molecular characterization and subsequent outbreak investigations. Although occult blood tests did not enhance the sensitivity of STEC recovery, patient data and accompanying epidemiologic information may help identify which samples to test, thereby preventing future outbreaks. Because such epidemiologic information is often not available to laboratory personnel, we suggest that clinical laboratories work with medical administrations to use EIAs as their standard of practice and to facilitate routine availability of such information. Until more sensitive and cost-effective STEC screening methods are available, facilities that cannot implement EIAs should forward stool samples that are suspect, as well as those with positive screening results, to public health laboratories. These laboratories can easily evaluate suspect stools for STEC by EIA or PCR followed by culture of all positive samples to recover the isolate for further characterization.
